# Thyroid Scintigraphy Findings in 234 Hyperthyroid Cats Before and After Radioiodine Treatment

**DOI:** 10.3390/ani15101495

**Published:** 2025-05-21

**Authors:** Lisa Stammeleer, Pilar Xifra, Sara I. Serrano, Eva Vandermeulen, Sylvie Daminet, Mark E. Peterson

**Affiliations:** 1 Small Animal Department, Faculty of Veterinary Medicine, Ghent University, 9820 Merelbeke, Belgium; 2 Pride Veterinary Referrals, Derby DE24 8HX, UK; 3 Iodocat, Leganés, 28919 Madrid, Spain; info@iodocat.es (P.X.);; 4 Department of Medical Imaging of Domestic Animals, Ghent University, 9820 Merelbeke, Belgium; vandermeuleneva@hotmail.com; 5 AniCura Hond en Kat, 9800 Deinze, Belgium; 6 Animal Endocrine Clinic, New York, NY 10025, USA; 7 Cornell University Hospital for Animals, Cornell University, Ithaca, NY 14850, USA

**Keywords:** hyperthyroidism, hypothyroidism, ^99m^Tc-pertechnetate, radioactive iodine, ^131^I

## Abstract

Thyroid scintigraphy is a key tool for diagnosing and staging hyperthyroidism in cats, but follow-up scintigraphic studies after radioiodine treatment are limited. This multicentric study evaluated ^99m^Tc-pertechnetate scintigraphy findings in 234 hyperthyroid cats before and 6 months after radioiodine treatment. Based on serum T4 and TSH concentrations, 165 (70.5%) became euthyroid, 54 (23.1%) had subclinical hypothyroidism, and 15 (6.4%) developed overt hypothyroidism. On post-treatment scintigraphy, all cats showed reduced size and radionuclide uptake of “hot” thyroid nodules. Of 99 cats with unilateral nodules, 60 (61%) recovered function in the contralateral lobe. Among 135 cats with bilateral nodules, both lobes remained visible in 108 (80%). Persistent “hot” nodules with high thyroid/salivary (T/S) ratios or thyroidal pertechnetate uptake (TcTU) occurred in 26 (11%) cats, all of which were euthyroid. Conversely, 24 (10.4%) cats had minimal or absent thyroid tissue with 17 (71%) being hypothyroid, but seven (29%) were euthyroid. As a diagnostic test for iatrogenic hypothyroidism, TcTU showed the highest sensitivity (62.3), with the T/S ratio (7.3) and background-corrected T/S ratio (30.4) being much lower (*p* < 0.01). While follow-up scintigraphy aids in assessing thyroid tumor destruction and residual function, its diagnostic utility for differentiating euthyroidism and hypothyroidism is limited, especially for cats with mild (subclinical) hypothyroidism.

## Simple Summary

Thyroid scintigraphy is an important tool for diagnosing and staging hyperthyroidism in cats, but it has not been evaluated for treatment success after radioiodine. In a study of 234 hyperthyroid cats, thyroid scans were performed before and six months after treatment. Based on thyroid blood tests, 165 cats (70.5%) returned to normal thyroid function, 54 cats (23.1%) had mild thyroid underactivity (subclinical hypothyroidism), and 15 cats (6.4%) developed more severe hypothyroidism. With follow-up scintigraphy, all cats showed a reduction in the size and activity of the overactive (“hot”) thyroid tumor tissue. In cats with one-sided thyroid disease, the opposite thyroid lobe regained function in 60 of 99 cats (61%). In cats with both thyroid lobes affected, 108 of 135 (80%) maintained thyroid function in both lobes. Persistent “hot” nodules occurred in 26 (11%) cats, but all had normal thyroid function. In contrast, 24 cats (10.4%) had little to no remaining thyroid tissue on follow-up scans, and most of these cats (17/24) had become hypothyroid. Although thyroid scans can help monitor the effectiveness of treatment and confirm destruction of the cat’s thyroid tumor, their ability to distinguish between normal and mildly underactive thyroid function is limited.

## 1. Introduction

Thyroid scintigraphy is widely recognized as the gold standard for diagnosing hyperthyroidism in cats [1,2,3,4,5]. Beyond diagnosis, scintigraphy plays a vital role in staging of the thyroid tumor disease and determining the optimal dose of radioiodine (^131^I) for treatment [6,7]. Following ^131^I treatment, cats may achieve euthyroidism, develop iatrogenic hypothyroidism (subclinical or overt subtypes), or less commonly, remain hyperthyroid [6,7,8,9,10,11,12,13]. Despite thyroid scintigraphy’s established utility, its role in evaluating post-treatment outcomes remains largely unexplored.

The ideal outcome after radioiodine therapy is euthyroidism, characterized by normal serum concentrations of both thyroxine (T4) and thyroid stimulating hormone (TSH) [1,6,7,9,10,11]. In hyperthyroid cats, the aim of ^131^I therapy is to selectively destroy all of the hyperfunctional, adenomatous thyroid nodules, whether unilateral, bilateral, and ectopic [13]. However, few studies have employed thyroid scintigraphy to reevaluate euthyroid cats previously treated with ^131^I [8,12]. Treatment failure or persistent hyperthyroidism is reported in 2–8% of cats treated with ^131^I [6,7,9,10,11,14,15]. Thyroid scintigraphy is valuable for confirming persistent hyperthyroidism, as well as determining the number, size, and location of residual thyroid tumor nodules after radioiodine treatment, similar to its role in initial diagnosis and staging of untreated hyperthyroid cats [9,16].

Iatrogenic hypothyroidism is well-recognized as a relatively common outcome in cats treated with ^131^I [6,7,8,9,10,11,17,18,19]. Identifying cats with ^131^I-induced hypothyroidism that also develop azotemic chronic kidney disease (CKD) is particularly critical, as this combination shortens survival time [8,17,19,20]. In untreated hyperthyroid cats, pre-treatment quantitative and qualitative scintigraphic findings, such as symmetrical bilateral disease with homogeneous radionuclide uptake into both lobes, have been associated with an increased risk of developing iatrogenic hypothyroidism [9,21,22]. In addition, thyroid scintigraphy is a useful diagnostic test for cats with congenital or spontaneous adult-onset hypothyroidism. [5,23,24] However, the role of thyroid scintigraphy for diagnosing and staging iatrogenic hypothyroidism cats after ^131^I treatment has not yet determined.

In this study, qualitative and quantitative thyroid scintigraphy findings are described in a large cohort of hyperthyroid cats before and after successful treatment with ^131^I. Furthermore, we sought to compare these scintigraphy results with serum T4 and TSH concentrations to evaluate the role of imaging in determining thyroid status, especially in cats with persistent hot thyroid nodules or iatrogenic hypothyroidism.

## 2. Materials and Methods

### 2.1. Study Population and Design

In this prospective before–after study of 234 hyperthyroid cats, data were collected from the following three ^131^I treatment centers: the Animal Endocrine Clinic in New York, Iodocat in Madrid, and the Small Animal Department at Ghent University. We evaluated hyperthyroid cats presented between 2014 and 2023, in which quantitative and qualitative thyroid scintigraphy was performed before and 6 months after ^131^I treatment (using previously described ^131^I dosing protocols) [6,7,12].

Cats were excluded from this study if they had received methimazole within 7 days of evaluation, if they had large multifocal or metastatic thyroid disease (e.g., suspected carcinoma or SHIM-RAD tumors) consistent with thyroid carcinoma [4,5,25], or if they remained persistently hyperthyroid. Ethics approvals were obtained from each Institution’s Animal Care and Use Committee. All owners provided written informed consent.

All cats underwent evaluations before and after treatment that included a review of medical history, complete physical examination, routine laboratory testing (CBC, serum biochemical profile, complete urinalysis) and determination of serum thyroid hormone concentrations (T4 and TSH) prior to and 6 months after ^131^I treatment. All thyroid (including free T4 concentrations, when needed) and TSH testing was conducted by assays validated for use in cats, as previously described [18]. To maintain consistency among the three study sites, all laboratory samples were submitted to a designated reference veterinary diagnostic laboratory (IDEXX Laboratories, Westbrook, ME, USA).

Based on serum creatinine concentrations at the 6-month recheck time, we classified the cats as non-azotemic vs. azotemic (serum creatinine > 2.0 mg/dL). We also classified the cats as being euthyroid vs. hypothyroid after ^131^I treatment, based on the following criteria: euthyroid (T4, 1.0–3.8 μg/dL; TSH ≤ 0.30 ng/mL), overtly hypothyroid (T4 < 1.0 μg/dL; TSH > 0.30 ng/mL), and subclinically hypothyroid (T4, 1.0–2.0 μg/dL; TSH > 0.30 ng/mL), as previously described [6,7,8,9,18].

### 2.2. Thyroid Scintigraphy

Qualitative and quantitative thyroid scintigraphy (before and after ^131^I treatment) was performed by injecting 111–185 MBq (3–5 mCi) of sodium 99mTc-pertechnetate (^99m^TcO_4_^−^) intravenously or subcutaneous and imaging 20–60 min later, as previously described [3,4]. All thyroid images were analyzed independently both by L.S. and M.E.P.

#### 2.2.1. Pre-Treatment Scintigraphy

**Figure 1:**
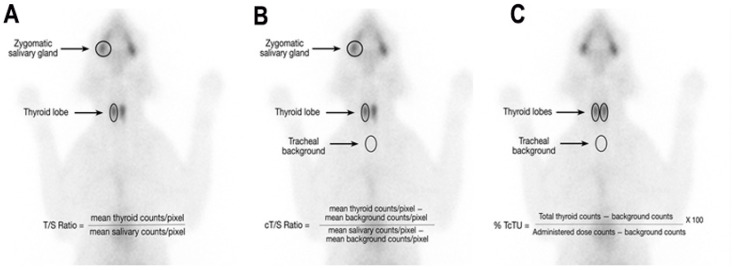
Drawing region of interest (ROIs) for calculation of (**A**) T/S ratio; (**B**) background-corrected T/S (cT/S); and (**C**) percent TcTU.

In untreated hyperthyroid cats, qualitative analysis allowed us to classify cats into 1 of 4 patterns of thyroid disease—unilateral, bilateral asymmetric in size, bilateral symmetrical in size, and ectopic-multifocal disease (≥3 areas or nodules of increased radionuclide uptake), as well as to help exclude thyroid carcinoma [3,4,5]. Quantitative analysis was used to calculate the thyroid-to-salivary gland ratio (T/S) and percent thyroidal uptake of the injected sodium ^99m^Tc-pertechnetate (TcTU), as previously described [3,4,5] (see Figure 1).

#### 2.2.2. Post-Treatment Scintigraphy

After ^131^I treatment, all cats again underwent both qualitative and quantitative thyroid scintigraphy. Qualitative analysis evaluated the number and size of each thyroid nodule, as well as each nodule’s intensity of radionuclide uptake (degree of brightness or hotness).

For quantitative analysis, the average T/S ratio was calculated by drawing a region of interest (ROI) around each thyroid lobe and respective zygomatic salivary gland, and then dividing the average thyroid count density (counts/pixels) of each thyroid lobe by the average salivary gland count density of each salivary gland, respectively (Figure 1A). If no clear thyroid or salivary tissue could be identified, a ROI was drawn in the region of the tissue’s pre-treatment location. In addition, an average, background-corrected T/S (cT/S) was also calculated by drawing a ROI around each thyroid lobe and salivary gland, as well as drawing a ROI just caudal to the thyroid midline over the trachea to obtain a “tracheal” background count density (Figure 1B) [4,8]. The cT/S ratio was calculated by dividing the thyroid count density (minus the tracheal background count density) by the salivary count density (minus the tracheal background count density) (Figure 1B) [8]. Finally, the percent TcTU was calculated by dividing the total thyroid counts by the injected ^9m^Tc-pertechnetate dose counts (Figure 1C), corrected for decay and depth corrected as previously described [4].

### 2.3. Data and Statistical Analysis

Data were assessed for normality by the D’Agostino–Pearson test and by visual inspection of graphical plots [26]. Data were not normally distributed; therefore, all analyses used were performed using nonparametric tests. Results are reported as the median and interquartile range (IQR; 25th–75th percentile) and are represented graphically as box plots [27]. For all analyses, statistical significance was defined as *p* ≤ 0.05.

We established reference intervals for quantitative scintigraphy parameters by imaging 190 clinically normal, euthyroid cats (>7 years of age; both sexes). Using a nonparametric method, we determined the central 95th percentile interval (2.5th to 97.5th percentile range) [28] and calculated 90% confidence intervals (CI) for the lower and upper limits of each reference interval (Table 1).

Results for qualitative (categorical) data are expressed as ratio (e.g., breed, sex) or percent of cats (e.g., unilateral disease, bilateral disease). Categorical variables were compared between groups by Chi-square or Fisher’s exact tests. Comparisons between continuous variables between 2 groups or within groups (before–after) were analyzed with the Mann–Whitney U test and Wilcoxon signed ranks test, respectively. Comparisons between 3 continuous variables were analyzed with the Kruskal–Wallis test followed by the Conover’s multiple comparisons test.

Finally, the diagnostic performance of 3 quantitative thyroid scintigraphy parameters (T/S ratio, cT/S ratio, and TcTU) for identifying cats with subclinical and overt hypothyroidism was evaluated. Test sensitivity and specificity were then calculated for each quantitative parameter [29]. The McNemar test [30] was used to determine whether differences existed between the sensitivity and specificity for T/S ratio, cT/S ratio, and TcTU as diagnostic tests for iatrogenic hypothyroidism.

Statistical analyses were performed using proprietary statistical software (GraphPad Prism, version 10.4; GraphPad Software, La Jolla, CA, USA; MedCalc, version 20.1, MedCalc Statistical Software, Ltd., Ostend, Belgium).

## 3. Results

### 3.1. Pre-Treatment Characteristics of Hyperthyroid Cats

This study enrolled 234 cats: 136 cats from Clinic 1 (New York), 62 from Clinic 2 (Madrid), and 36 from Clinic 3 (Ghent). The 234 cats ranged in age from 5 to 20 years (median, 12 years; Table S1). Breeds included Domestic Shorthair and Longhair (*n* = 213; 91%), Siamese (7), Norwegian Forest Cat (3), Ragdoll (2), Persian (2), British Shorthair (1), Maine Coon (1), Burmese (1), American Curl (1), Bombay (1), Devon Rex (1), and one Scottish Fold. Of the 234 cats, 131 (56%) were female and 103 cats (44%) were male; 231 cats (99%) were neutered, and three cats were intact.

Of the hyperthyroid cats, 229/234 (98%) had high serum T4 concentrations (median 9.2 μg/dL, Table S1). The five cats with normal serum T4 concentrations had high serum free T4 concentrations, as well as scintigraphic evidence showing “hot” thyroid nodules, with high TcTU and thyroid-to-salivary gland ratios. Serum TSH concentrations were below the limit of detection (<0.03 ng/mL) in 226/234 cats (97%). Of the five cats with normal serum T4 concentrations, two cats had measurable TSH concentrations (0.04 and 0.06 ng/mL). The median serum creatinine concentration was 1.0 mg/dL (Table S1).

Hyperthyroid cats from the three treatment sites showed no differences in age, breed, or sex distribution. Similarly, serum concentrations of creatinine, T4, and TSH did not differ among cats from the three sites.

### 3.2. Pre-Treatment Thyroid Scintigraphy in Hyperthyroid Cats

Of the 234 hyperthyroid cats, 135/234 (57.7%) cats had bilateral thyroid gland disease (i.e., two “hot” thyroid nodules), while 99/234 (42.3%) cats had unilateral thyroid gland disease (i.e., one “hot” thyroid nodule) and 5/234 (2.1%) cats had ectopic thyroid gland disease. Among the ectopic cases, four cats had ectopic thyroid tissue in combination with unilateral right-sided disease, and one cat had ectopic thyroid tissue in combination with bilateral disease (Table S1).

Of the 135 cats with bilateral disease, 108 (80%) had bilateral asymmetric thyroid gland disease, whereas 27 (20%) cats had bilateral symmetric thyroid disease. Of the 99 cats with unilateral disease, 45 (45.5%) cats had left-sided thyroid nodules and 54 (54.5%) cats had right-sided disease.

With quantitative thyroid scintigraphy, 227/234 (97%) cats had high TcTU values, while 7/234 (3%) had values within the upper end of the reference interval (0.70–0.91%). Similarly, 229/234 (98%) of the hyperthyroid cats had high T/S ratios, with only two (2%) having ratios within the reference interval of <1.5 (1.0–1.24; Table S1).

### 3.3. Dose of Radioiodine Administered to Treat Hyperthyroid Cats

The 234 hyperthyroid cats were treated with ^131^I doses, administered subcutaneously, ranging from 1.2 mCi (3.6 MBq) to 12 mCi (444 MBq), with a median dose of 2 mCi (74 MBq) (Table S1). Of these, only 51/234 cats (22%) received doses greater than 2.5 mCi (90 MBq), and 35/234 cats (15%) received doses greater than 3 mCi (110 MBq).

### 3.4. Post-Treatment Serum Concentrations of T4, TSH and Creatinine

After ^131^I treatment, the 234 cats were re-evaluated after a median time of 6.8 months (IQR, 6.1–7.5 months). Treated cats had a decrease in serum T4 concentration, falling from 9.2 µg/dL to 1.7 μg/dL; *p* < 0.001; Table 2 and Table 3). These treated cats also had an increase in serum TSH, rising from undetectable concentrations (<0.03 ng/mL) to 0.11 ng/mL (*p* < 0.001; Table S1 and Table 2).

**Table 2:** Post-treatment serum T4, TSH, and creatinine concentrations and quantitative thyroid scintigraphic findings in 234 ^131^I-treated cats, divided into three groups based on thyroid status.

Variable	All Cats (234)	Euthyroid (165)	Overt Hypothyroid (15)	Subclinical Hypothyroid (54)	*p* Value
Serum creatinine (mg/dL)	1.6 (1.3–1.9)	1.5 ^a,b^ (1.3–1.8)	2.1 ^a,c^ (1.7–2.6)	1.7 ^b,c^ (1.4–2.1)	<0.0001
Serum T4 (µg/dL)	1.7 (1.3–2.1)	1.9 ^a,b^ (1.6–2.3)	0.8 ^a,c^ (0.6–0.9)	1.4 ^b,c^ (1.1–1.7)	<0.0001
Serum TSH (ng/mL)	0.11 (0.04–0.48)	0.07 ^a,b^ (0.03–0.13)	6.2 ^a,c^ (3.6–12.0)	0.88 ^b,c^ (0.52–2.45)	<0.0001
TcTU (%)	0.32 (0.18–0.59)	0.37 ^a,b^ (0.26–0.65)	0.14 ^a^ (0.06–0.19)	0.17 ^b^ (0.13–0.34)	<0.0001
T/S ratio	0.84 (0.68–1.05)	0.90 ^a,b^ (0.75–1.13)	0.60 ^a,c^ (0.48–0.79)	0.74 ^b,c^ (0.63–0.98)	<0.0001
cT/S ratio	0.73 (0.46–1.15)	0.81 ^a,b^ (0.56–1.25)	0.26 ^a,c^ (0.11–0.44)	0.51 ^b,c^ (0.34–0.98)	<0.0001

TcTU, percent thyroidal uptake of ^99m^TcO_4_; T/S, average thyroid-to-salivary ratio; cT/S, average background-corrected thyroid-to-salivary ratio. Superscript letters indicate significant differences (*p* ≤ 0.05).

Based on serum T4 and TSH concentrations, 165/234 (70.5%) cats became euthyroid, 15/234 (6.4%) cats developed overt hypothyroidism, and 54/234 (23.1%) cats had subclinical hypothyroidism. The 69 cats with subclinical or overt hypothyroidism had lower serum T4 and higher TSH concentrations than did euthyroid cats (*p* < 0.001; Table 2). Cats with overt hypothyroidism had lower serum T4 and higher TSH concentrations than did cats with subclinical hypothyroidism (*p* < 0.001; Table 2).

Additionally, ^131^I-treated cats had an increase in serum creatinine concentration, rising from 1.0 to 1.6 mg/dL (Table S1 and Table 2). Forty-eight cats, 48 (20.5%) developed high serum creatinine (>2 mg/dL) concentrations after treatment. Cats with subclinical or overt hypothyroidism had higher serum creatinine concentrations than did euthyroid cats (*p* < 0.001; Table 2).

### 3.5. Follow-Up Thyroid Scintigraphy After Treatment with ^131^I

**Figure 2:**
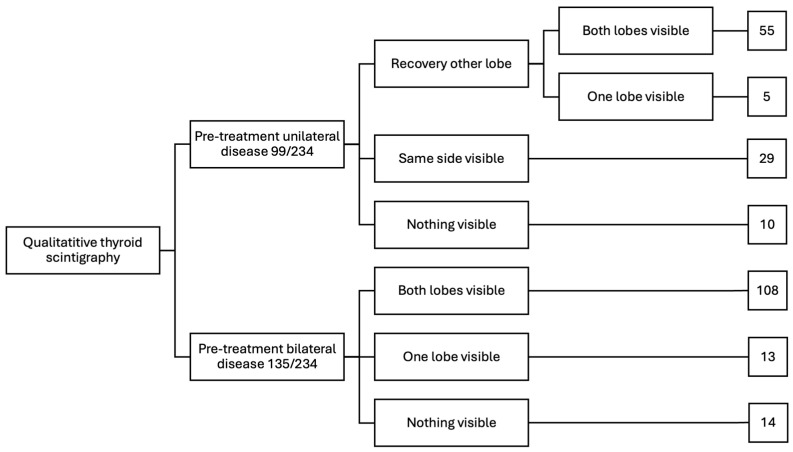
Results of follow-up scintigraphy in 234 hyperthyroid cats treated with radioiodine.

**Figure 3:**
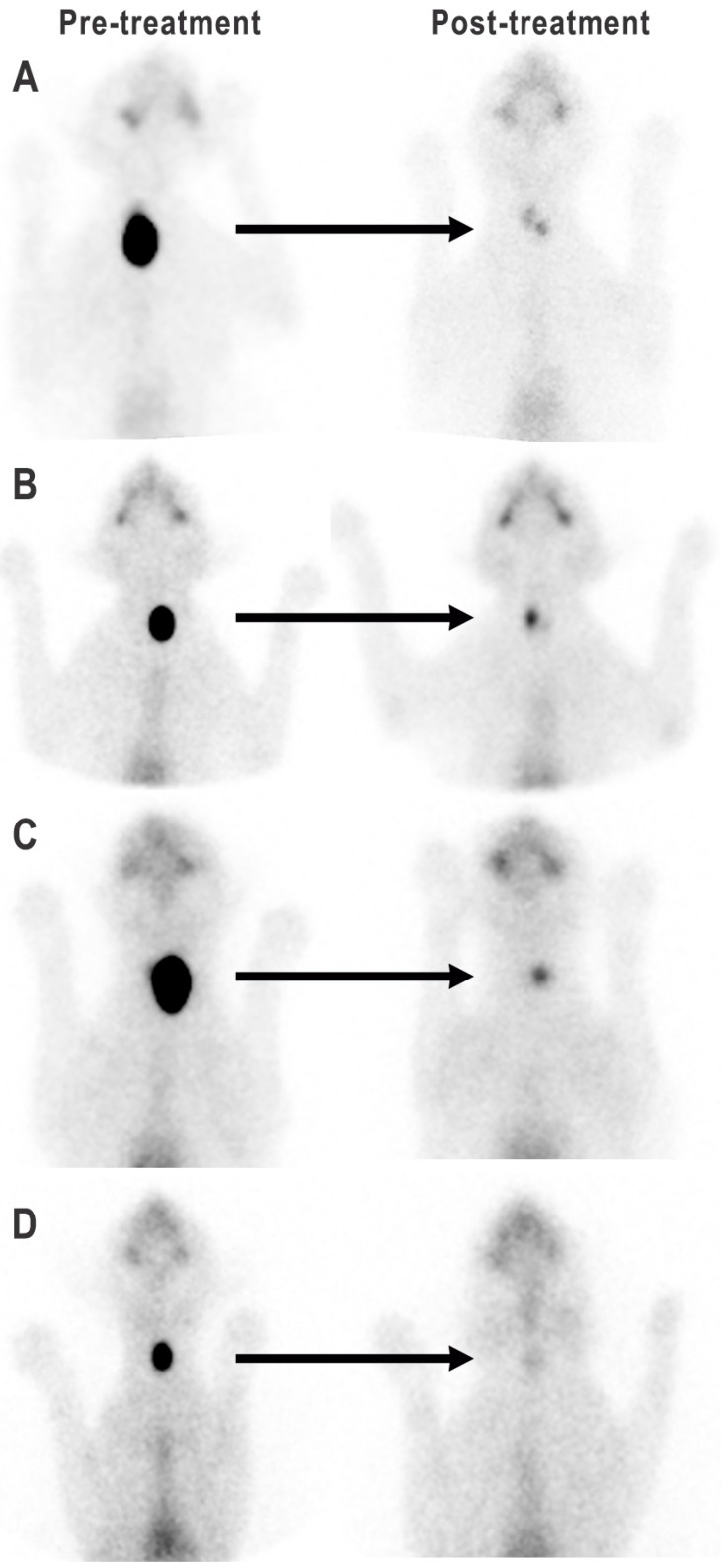
Thyroid scintigraphy in four cats with unilateral thyroid nodules, before and after treatment with ^131^I. (**A**) Reduction in size and radionuclide uptake of the “hot” right-sided thyroid nodule, with recovery of the previously suppressed left thyroid lobe; (**B**) Complete ablation of unilateral left thyroid nodule, with recovery of the contralateral right lobe; (**C**) Reduction in size and uptake intensity of the left thyroid nodule, but the contralateral right lobe remains suppressed. (**D**) Complete ablation of both the hot nodule and normal contralateral lobe.

**Figure 4:**
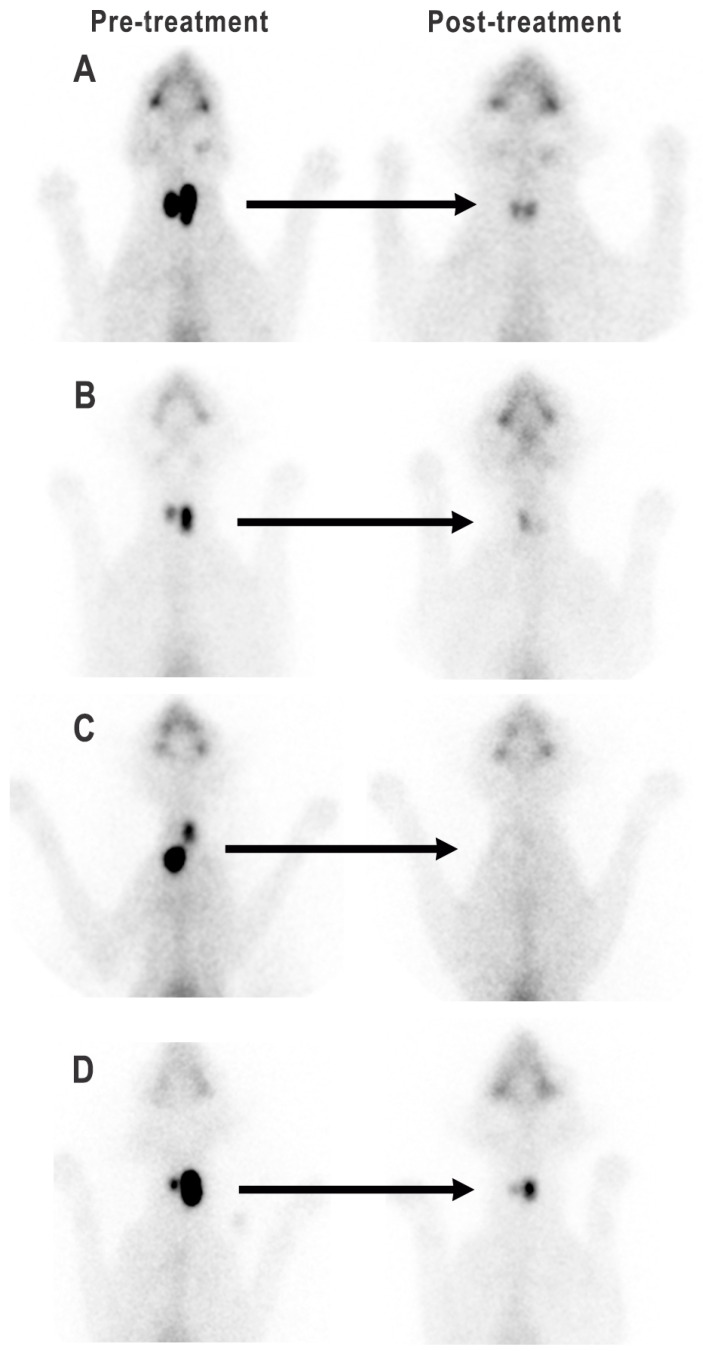
Thyroid scintigraphy in four cats with bilateral thyroid nodules, before and after treatment with ^131^I. (**A**) Reduction in size and radionuclide uptake of both “hot” thyroid nodules; (**B**) Complete ablation of the left thyroid nodule, with preservation of right thyroid lobe; (**C**) Near complete ablation of both thyroid nodules; (**D**) Reduction in size and radionuclide uptake of both thyroid nodules, with a persistent left-sided “hot” nodule.

After radioiodine treatment, all 234 cats had reduction in the size and brightness (i.e., intensity of radionuclide uptake) of their “hot” thyroid tumor nodule(s) as assessed by qualitative thyroid scintigraphy (Figure 2, Figure 3 and Figure 4). However, 26/234 cats (11%) retained a small “hot” nodule, while 24/234 ^131^I-treated cats (10.3%) had little to no visible thyroid tissue on follow-up thyroid scintigraphy (Figure 2, Figure 3 and Figure 4).

Of the 99 hyperthyroid cats with unilateral thyroid nodules, 60 cats (61%) had recovery of the contralateral, previously suppressed normal thyroid lobe (Figure 2 and Figure 3A,B). However, in five of these 60 cats, the primary hot unilateral nodule was completely destroyed and only the contralateral, previously suppressed lobe was visible (Figure 3B). In 29/99 cats (29%) with unilateral tumors, the contralateral side did not recover, and only the initially affected lobe remained visible (Figure 2 and Figure 3C). The remaining 10 cats (10%) with unilateral thyroid nodules had little to no visible thyroid tissue on scintigraphy after ^131^I treatment (Figure 2 and Figure 3D).

In 108 (80%) of the 135 cats with bilateral thyroid nodules, both lobes remained visible but were reduced in size and had lower intensity of uptake (i.e., brightness) (Figure 2 and Figure 4A). In 13 cats (9.6%) one lobe was completely destroyed, leaving only a single contralateral lobe visible (Figure 2 and Figure 4B). In none of the five cats with initial detected ectopic thyroid tissue could ectopic nodules be visualized after ^131^I treatment. The remaining 14 cats (10.4%) with bilateral thyroid nodules had little to no visible thyroid tissue on scintigraphy after ^131^I treatment (Figure 2 and Figure 4C).

**Figure 5:**
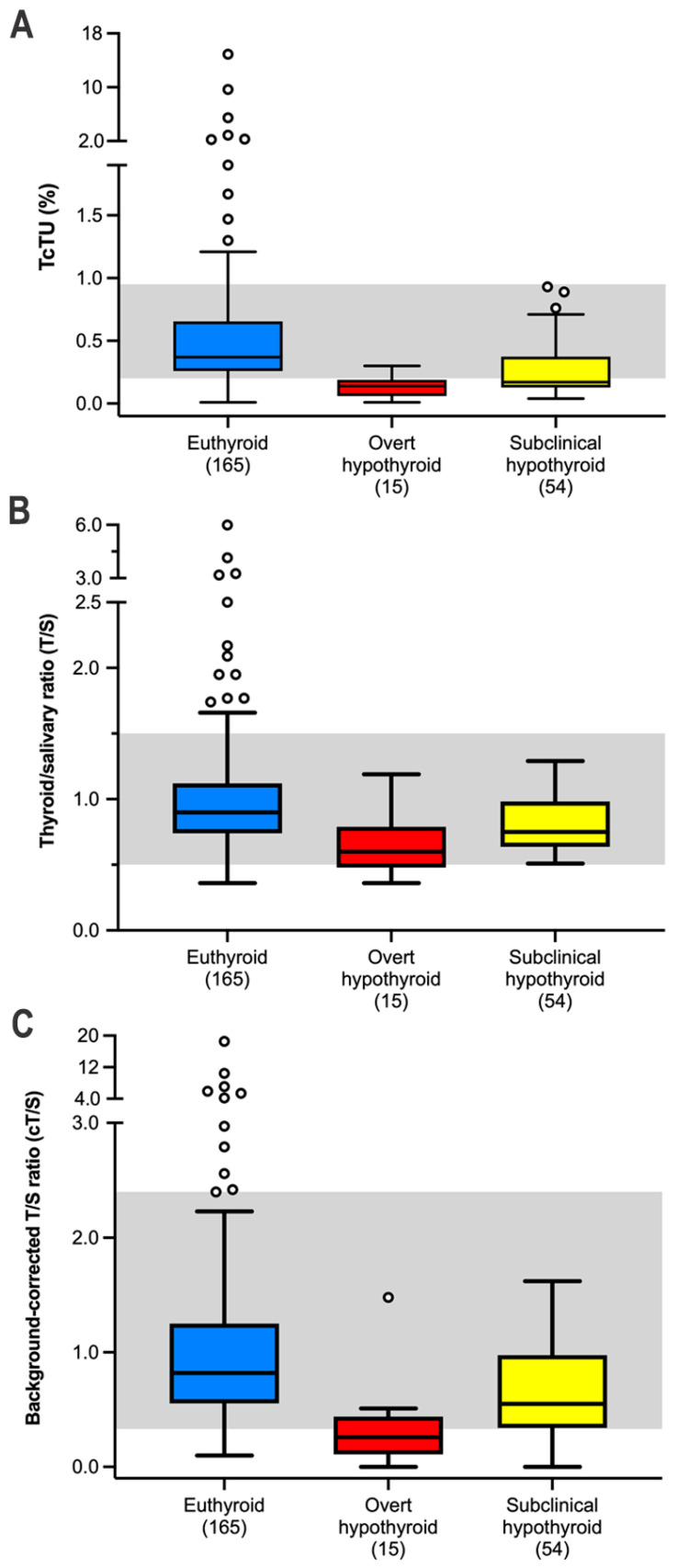
Box plots of follow-up quantitative scintigraphic results in 234 cats, divided into the 165 euthyroid cats and 69 with overt and subclinical hypothyroidism. (**A**) TcTU; (**B**) T/S ratio; and (**C**) background-corrected T/S ratio. Boxes represent the interquartile range from the 25th to 75th percentile. The horizontal bar in each box represents the median value. The T-bars represent the main body of data, which in most instances is equal to the range. Outlying data points are represented by open (white) circles. The shaded areas represent the reference intervals.

With quantitative thyroid scintigraphy, the 234 ^131^I-treated cats had decreases in percent TcTU and T/S ratios (Table 2 and Table 3). Euthyroid cats had higher values for TcTU, T/S ratio, and cT/S ratio than did cats with subclinical or overt hypothyroidism (Table 3, Figure 5). Among the 69 hypothyroid cats, the 15 cats with overt hypothyroidism had lower T/S and cT/S ratios than did cats with subclinical hypothyroidism (Table 3, Figure 5).

### 3.6. Cats with a Persistent Hot Thyroid Nodule on Scintigraphy After ^131^I Treatment

Twenty-six (11%) of the 234 ^131^I treated cats had a persistent hot thyroid nodule on follow-up thyroid scintigraphy (Figure 4D). All 26 of these cats were euthyroid based on serum T4 and TSH concentrations. Of these, 22 (85%) cats had a solitary hot thyroid nodule, whereas four had bilateral thyroid nodules. All 26 cats with persistent hot thyroid nodules had high values for TcTU, T/S ratio, or cT/S ratio on quantitative scintigraphy (Figure 5, Table 3). None of the hypothyroid (overt or subclinical) ^131^I-treated cats had a persistent hot thyroid nodule or high values for TcTU, T/S ratio, or cT/S ratio.

**Table 3:** Radioiodine dose, pre- and post-treatment serum T4 and TSH concentrations, and quantitative scintigraphic findings in 26 ^131^I-treated cats with a persistent thyroid nodule compared to 208 cats without thyroid nodules on follow-up scintigraphy.

Variable	Persistent Hot Nodule (26)	No Hot Nodule (208)	*p* Value
Dose radioiodine (mCi)	2.15 (1.8–2.5)	2.0 (1.8–2.5)	0.39
Pre-treatment serum T4 (µg/dL)	11.7 (8.7–12.6)	8.9 (6.6–12.4)	0.099
Pre-treatment serum TSH (ng/mL)	0.02 (0.02–0.02)	0.02 (0.02–0.02)	0.82
Pre-treatment T/S ratio	8.55 (5.8–11.4)	5.0 (3.1–8.7)	0.0006
Pre-treatment TcTU (%)	6.9 (3.7–11.7)	3.6 (2.2–7.5)	0.004
Post-treatment serum T4 (µg/dL)	2.3 (1.9–3.1)	1.7 (1.2–2.1)	<0.0001
Post-treatment serum TSH (ng/mL)	0.02 (0.02–0.04)	0.14 (0.06–0.61)	<0.0001
Post-treatment T/S ratio	2.0 (1.8–3.2)	0.82 (0.7–1.0)	<0.0001
Post-treatment TcTU (%)	1.96 (1.61–3.27)	0.64 (0.43–0.99)	<0.0001

All data listed as the median, with the Interquartile range (25th–75th percentile) in parentheses. Before and after data analyzed with the Mann–Whitney test. Reference intervals: T4 = 1.0–3.8 μg/d; TSH ≤ 0.03–0.3 ng/mL; T/S ratio = 0.5–1.5; TcTU = 0.2–0.95% (see Table 1).

When the 26 cats with persistent hot nodules were compared to the other 208 cats, the administered ^131^I dosage did not differ between the two groups (Table 4). Similarly, pre-treatment serum T4 and TSH concentrations were comparable between the groups (Table 3). However, the 26 cats with persistent hot nodules had higher pre-treatment values for T/S ratio and TcTU compared to the 208 cats without hot nodules (Table 4).

After ^131^I treatment, the 26 cats with a persistent hot thyroid nodule had higher post-treatment serum T4 concentrations and lower post-treatment serum TSH concentrations than did 208 cats without a persistent nodule (Table 4). In addition, the 26 cats with persistent hot nodules had higher values for T/S ratio and TcTU compared with the 208 cats without hot nodules (Table 3). All 26 cats had a high T/S ratio, whereas 13 (50%) had high TcTU values.

Long-term follow-up was available in 17/26 cats with a persistent hot thyroid nodule (median follow-up, 36.1 months, IQR 24.5–50.4 months). Among these 17 cats, 14 (82%) cats remained euthyroid, 2 (12%) became subclinical hypothyroid, and one cat (6%) experienced a relapse of hyperthyroidism.

### 3.7. Cats with Little to No Visible Thyroid Tissue on Scintigraphy After ^131^I Treatment

Twenty-four (10.3%) of the 234 cats had little to no thyroid tissue visible on scintigraphy after ^131^I treatment. Prior to treatment, 10 (42%) of these 24 cats had unilateral thyroid disease, while 14 (58%) had bilateral thyroid disease (Figure 3D and Figure 4C).

Based on serum T4 and TSH measurements, 17/24 (71%) of these cats were hypothyroid, and 7/24 (29%) were euthyroid. Of the 17 hypothyroid cats, 10/17 (59%) cats were subclinically hypothyroid and 7/17 (41%) were overtly hypothyroid. The 24 cats with no visible thyroid tissue on scintigraphy were more likely to be hypothyroid than the 210 cats that had clearly visible thyroid tissue (17/24 [71%] vs. 52/210 [25%]; *p* < 0.001).

When the 24 cats with little to no visible thyroid tissue on scintigraphy were compared to the other 210 cats in which thyroid tissue could clearly be identified, the administered ^131^I dosage did not differ between the two groups (Table 4). Similarly, pre-treatment serum T4 and TSH concentrations, T/S ratios, and TcTUs were comparable between the groups (Table 4).

After ^131^I treatment, the 24 cats with little to no visible thyroid tissue had lower post-treatment serum T4 concentrations and higher post-treatment serum TSH concentrations compared with the 210 cats in which thyroid tissue could clearly be identified (Table 5). In addition, the 24 cats with little to no visible thyroid tissue had lower values for T/S ratio and TcTU than did the 210 cats in which thyroid tissue could clearly be identified (Table 4).

**Table 4:** Pre- and post-treatment serum T4 and TSH concentrations and quantitative scintigraphic findings in 24 ^131^I-treated cats with little to no visible thyroid tissue on scintigraphy compared to 210 cats with visible thyroid tissue on follow-up scintigraphy.

Variable	Little Visible Thyroid Tissue (24)	Clearly Visible Thyroid Tissue (210)	*p* Value
Dose radioiodine (mCi)	2.1 (1.8–2.95)	2.0 (1.8–2.5)	0.62
Pre-treatment serum T4 (µg/dL)	9.1 (6.9–11.8)	9.2 (6.6–12.5)	0.84
Pre-treatment serum TSH (ng/mL)	0.02 (0.02–0.02)	0.02 (0.02–0.02)	0.92
Pre-treatment T/S ratio	4.2 (3.0–6.7)	5.5 (3.5–9.1)	0.22
Pre-treatment TcTU (%)	2.8 (2.0–3.9)	3.6 (2.1–7.7)	0.09
Post-treatment serum T4 (µg/dL)	1.05 (0.9–1.4)	1.8 (1.4–2.2)	<0.0001
Post-treatment serum TSH (ng/mL)	1.20 (0.1–0.3)	0.10 (0.04–0.32)	<0.0001
Post-treatment T/S ratio	0.2 (0.1–0.35)	0.8 (0.5—1.2)	<0.0001
Post-treatment TcTU (%)	0.08 (0.04–0.12)	0.34 (0.22–0.63)	<0.0001

All data listed as the median and interquartile range (25th–75th percentile). Before and after data analyzed with the Mann–Whitney test. Reference intervals: T4 = 1.0–3.8 μg/d; TSH ≤ 0.03–0.3 ng/mL; T/S ratio = 0.5–1.5; TcTU = 0.2–0.95% (see Table 1).

Of the 24 cats with no visible thyroid tissue on post-treatment scintigraphy, 18 had long-term follow-up data (median, 30.6 months; IQR, 13.7–49.2 months). Thyroid status changed in only one of these 18 cats after the initial 6-month evaluation. This cat, initially subclinical hypothyroid, became euthyroid 75 months (6.3 years) after ^131^I treatment. All seven of the euthyroid cats remained euthyroid on long-term follow-up.

### 3.8. Sensitivity and Specificity of TcTU, aT/S Ratio and cT/S Ratio as Diagnostic Tests for Hypothyroidism in ^131^I-Treated Cats

When evaluated as a diagnostic test for iatrogenic hypothyroidism, TcTU demonstrated the highest diagnostic test sensitivity (62.3), with the cT/S ratio (30.4) and T/S ratio (7.3) having much lower values (*p* < 0.01). The diagnostic test sensitivity for all three parameters were higher in the 15 cats with overt hypothyroidism compared to the 54 cats with subclinical hypothyroidism (Table 5).

The TcTU, T/S ratio and cT/S ratio all exhibited relatively high test specificity, both in the treated euthyroid cats and the clinically normal cats (Table 5).

**Table 5:** Calculation of test sensitivity and specificity for TcTU, T/S ratio, and cT/S ratio as diagnostic tests for ^131^I-induced hypothyroidism in cats.

	Test Sensitivity (95% CI *)			Test Specificity (95% CI *)		
Parameter	Subclinical Hypothyroid (54)	Overt Hypothyroid (15)	All Hypothyroid (69)	Euthyroid Post-Treatment (165)	Clinically Normal (190)	All Euthyroid (355)
TcTU	55.6 (41.4–69.1)	86.7 (59.5–98.3)	62.3 (49.8–73.7)	85.5 (79.1–90.5)	98.9 (96.3–99.9)	93.1 (90.1–95.5)
T/S ratio	1.0 (0.03–5.5)	33.3 (11.8–61.6)	7.3 (2.4–16.1)	98.2 (94.8–99.6)	98.4 (95.5–99.7)	98.3 (96.4–99.4)
cT/S ratio	20.4 (10.6–33.5)	66.7 (38.4–88.2)	30.4 (19.9–42.7)	92.1 (86.9–95.7)	98.4 (95.5–99.7)	95.5 (92.8–97.4)

* CI = confidence interval.

## 4. Discussion

Our findings demonstrate that thyroid scintigraphy provides valuable insights into morphological and functional changes in the nodular thyroid gland after radioiodine treatment. All 234 treated cats showed reduction in size and radionuclide uptake of their hyperfunctional thyroid nodules, with three distinct post-treatment scintigraphic patterns emerging: visible bilateral or unilateral thyroid tissue with normal ^99m^Tc-pertechnetate uptake, persistent “hot” nodules, or minimal to absent thyroid tissue.

In hyperthyroid cats with unilateral disease, over 60% showed recovery of the previously suppressed contralateral lobe. In all these cats, high serum thyroid hormone concentrations normalized after treatment, which removed the negative feedback inhibition on pituitary thyrotropes associated with hyperthyroidism [31,32,33]. The subsequent rise in circulating TSH concentrations stimulated the dormant “normal” thyroid tissue to function again. In 30% of cats with unilateral tumors, however, the suppressed contralateral thyroid lobe did not become visible on follow-up scintigraphy. It is certainly possible that this contralateral suppressed thyroid lobe might have recovered later than our 6-month follow-up scintigraphy. It is more likely, however, that ^131^I ablated this normal lobe, as has also been reported in human patients [34]. Similarly, in the remaining 10% of cats with unilateral tumors that had little to no thyroid tissue visible on follow-up scintigraphy, the absence of visible tissue suggests ^131^I-induced destruction of both thyroid lobes. Theoretically, suppressed thyroid tissue might be expected to neither concentrate nor be destroyed by ^131^I [35], and it should resume function after ablation of the unilateral thyroid nodule [13,22]. Yet previous studies show that cats with unilateral thyroid nodules can develop overt hypothyroidism [9,20], indicating that ^131^I can potentially ablate normal thyroid tissue [9,22].

In hyperthyroid cats with bilateral disease, 80% maintained visible thyroid tissue in both lobes post-treatment, albeit with reduced size and uptake intensity, suggesting partial preservation of functional thyroid tissue. To understand how cats with bilateral thyroid disease can have hyperplastic or adenomatous nodules destroyed by ^131^I and remain euthyroid, one must know that feline hyperthyroid is caused by single or multiple hyperplastic-adenomatous nodules within the affected lobe that take up and concentrate ^131^I [36,37]. However, almost all of these cats also have clumps of normal, suppressed paranodular tissue within the affected lobe that do not concentrate ^131^I after treatment [36,37]. Therefore, after ^131^I treatment, these previously suppressed clumps of normal paranodular tissue will recover and maintain euthyroidism. In the remaining 20% of the cats with bilateral disease, half had one lobe completely destroyed, leaving only a single contralateral lobe visible, whereas the remaining cats had little to no visible thyroid tissue on follow-up scintigraphy after ^131^I treatment.

A notable finding in this study was the presence of persistent “hot” nodules in 26 (11%) of the 234 treated cats, all of which were euthyroid. This phenomenon, also reported in human patients after ^131^I treatment [34], highlights the fact that increased ^99m^Tc-pertechnetate uptake cannot always be used to predict thyroid hormone overproduction (hyperthyroidism). Although both ^99m^Tc-pertechnetate and iodine isotopes (e.g., ^131^I, ^123^I, ^127^I) are actively transported into thyroid cells via the sodium-iodine symporter (the iodine pump) [38], only iodine is organified and incorporated into thyroid hormones [39,40]. In human patients, hot nodules on ^99m^Tc-pertechnetate scintigraphy may appear warm or even cold with ^123^I imaging, suggesting preserved thyroid uptake function but impaired hormone synthesis in these nodules [41,42,43,44]. This mechanism may explain how all of our cats with persistent hot nodules were euthyroid at time of repeat scintigraphy and only one cat had a relapse of hyperthyroidism much later (at 31 months after initial treatment). Comparing ^99m^Tc-pertechnetate to ^123^I scans in these euthyroid cats would be of great interest to see if the nodules remained “hot” on ^123^I scintigraphy or if they would have a normal ^123^I uptake.

Although all of our treated cats with persistent hot thyroid nodules were euthyroid, they had lower serum TSH and higher serum T4 concentrations than our cats without a persistent hot nodule. This is similar to findings in ^131^I-treated euthyroid human patients shown to have hot thyroid nodules on follow-up scintigraphy [34,45,46]. Overall, these findings—increased T/S ratio, high TcTU, suppressed TSH concentrations, and higher T4 concentrations—provide strong evidence that these hot nodules continue to function autonomously (as in untreated hyperthyroid cats [36,37]), even after the cats become euthyroid. This autonomous function can also result in persistent suppression of the pituitary-thyroid axis, thereby preventing the recovery of the surrounding normal thyroid tissue.

Finally, 24 of our 234 cats (10%) showed minimal or absent thyroid tissue on scintigraphy. Of these, almost 60% had bilateral thyroid disease, while over 40% had unilateral nodules. In the cats with unilateral disease, a longer follow-up period may have been needed to see the suppressed normal tissue recover, but again, this normal tissue likely was also destroyed by the ^131^I.

Of the 69 hypothyroid cats in this study (54 with subclinical and 15 with overt disease), 17 (25%) had little to no thyroid tissue visible on scintigraphy, whereas some (but generally less-than-normal amounts of) thyroid tissue could be visualized in the remaining 52 cats. A greater proportion of cats with overt hypothyroidism (47%) had no visible thyroid tissue compared to cats with subclinical hypothyroid (18.5%), suggesting a correlation between the degree of thyroid destruction and severity of thyroid hypofunction. Since cats with subclinical hypothyroidism have milder disease and maintain low-normal serum T4 concentrations [6,8,9], it might not be all that surprising that over half of these subclinically hypothyroid cats had some thyroid tissue visible on qualitative scintigraphy. However, the presence of visible thyroid tissue in over half of cats with overt hypothyroidism is difficult to explain but indicates that scintigraphic appearance alone does not always reflect functional thyroid capacity. It appears that some hypothyroid cats may retain thyroid tissue capable of concentrating or taking up ^99m^Tc-pertechnetate but unable to produce or secrete normal amounts of thyroid hormone [47]. Similar findings have been documented in human patients, leading to the scientific consensus that thyroid scintigraphy has limited diagnostic utility in hypothyroidism [48,49].

Particularly intriguing was the finding that seven (4.2%) the 165 euthyroid cats in this study had little to no remaining thyroid tissue visible on scintigraphy. Similar observations in human patients suggest that while ^99m^Tc-pertechnetate uptake function may be compromised, sufficient iodine trapping capability (below the threshold detection by scintigraphy) remains to support adequate hormone production [50,51]. This discrepancy between scintigraphic appearance and thyroid function again emphasizes the limitations of pertechnetate scintigraphy in fully characterizing post-treatment thyroid status [48,49]. For research purposes, comparing ^99m^Tc-pertechnetate to ^123^I scans in these euthyroid cats would be of great interest to see if normal amounts of thyroid tissue were visible on ^123^I scintigraphy in these euthyroid cats.

Of the three quantitative scintigraphic variables evaluated (TcTU, T/S ratio, and background-corrected T/S ratio), TcTU proved to be the most reliable indicator of thyroid function after ^131^I treatment. While the T/S ratio is highly sensitive for diagnosing hyperthyroidism and correlates well with serum T4 concentrations in untreated hyperthyroid cats [3,52], its diagnostic utility appears more limited for evaluating post-treatment thyroid status, especially in cats with persistent hot nodules or for identifying cats with iatrogenic hypothyroidism.

In the 26 cats with persistent hot nodules of this study, all had high T/S ratio (a false-positive diagnostic result for hyperthyroidism), despite normal serum T4 concentrations and clinical euthyroidism. In contrast, only 13 (50%) of these cats had high TcTU values on follow-up quantitative scintigraphy. Therefore, finding a high T/S ratio or TcTU value after ^131^I treatment should not be equated to persistent hyperthyroidism or treatment failure, as only one cat experienced a relapse, and that occurred only many months after treatment.

For diagnosing iatrogenic hypothyroidism after ^131^I treatment, TcTU demonstrated the highest diagnostic test sensitivity (62.3%) compared with much poorer sensitivity for both T/S ratio (7.3%) and background-corrected T/S ratio (30.4%). The diagnostic sensitivity of TcTU was notably higher in cats with overt hypothyroidism (86.7%) compared to those with subclinical disease (55.6%), suggesting TcTU better identifies cats with more severe thyroid hypofunction. The poor diagnostic value of the post-treatment T/S ratio largely stems from the fact that background count density (counts/pixel) rarely measured less than half of the salivary count density, making a low calculated T/S ratio nearly impossible, even in cats with no visible thyroid tissue on scintigraphy. While background correction (cT/S) improved test sensitivity, it remained inferior to TcTU. All three parameters, however, exhibited high specificity (86–99%) in both treated euthyroid cats and clinically normal cats.

In agreement with our findings, TcTU provides the most accurate scintigraphic assessment of thyroid function in both humans and dogs with hypothyroidism [49,53,54]. Our results also agree with two other small studies of hypothyroid cats that had repeat scintigraphy after ^131^I treatment [8,12]. In the first study of 22 cats, TcTU correctly diagnosed four out of six (66.7%) cats with subclinical hypothyroidism, similar to the findings in this current study (55.6%) None of the 22 cats in the study had overt hypothyroidism [12]. In the second study of 28 hypothyroid cats that also had become azotemic after ^131^I treatment, TcTU was diagnostic in all 15 cats (100%) with overt hypothyroidism and in 10/13 cats (77%) with subclinical hypothyroidism, again similar to the results of this study.

This study has some limitations. First, long-term follow-up data were not available for all cats, limiting the ability to assess the predictive value of 6-month scintigraphy. Second, because ^123^I is both trapped and organified, inclusion of ^123^I scintigraphy may have clarified how some of our euthyroid cats could maintain a persistent hot thyroid nodule or have no visible thyroid tissue on ^99m^Tc-pertechnetate imaging. However, ^123^I has many disadvantages over ^99m^Tc-pertechnetate, including higher cost, longer half-life (13.2 vs. 6 h), longer time from injection of isotope to imaging time (24 h vs. 20–60 min), and longer acquisition times (thus the need for sedation to reduce motion in most cats).

## 5. Conclusions

In this study, follow-up thyroid scintigraphy after ^131^I treatment effectively documented reductions in tumor size and radionuclide uptake, along with the recovery of previously suppressed, normal thyroid tissue. However, specific scintigraphic patterns showed limited correlation with serum thyroid function. Persistent “hot” nodules did not predict hyperthyroidism, as all of our affected cats were euthyroid on serum T4 and TSH testing. Similarly, absence of visible thyroid tissue on follow-up scintigraphy was not always diagnostic for hypothyroidism, as this pattern was also seen in a few euthyroid cats. Of the quantitative scintigraphic measurements, percent thyroidal uptake (TcTU) proved most accurate for diagnosing post-treatment hypothyroidism, but TcTU only had a modest test sensitivity (62.3%).

These findings suggest that while follow-up scintigraphy provides valuable information about anatomic and functional changes after ^131^I therapy, serum thyroid hormone testing remains essential for determining the cats’ post-treatment thyroid status, particularly in cats with mild or subclinical hypothyroidism.

## Figures and Tables

**Table 1:** Reference intervals for percent TcTU, aT/S ratio, and cT/S ratio based on results from 190 clinically normal, euthyroid cats.

Variable	Median (IQR)	Lower Reference Limit (90% CI)	Upper Reference Limit (90% CI)
TcTU (%)	0.41 (0.32–0.55)	0.20 (0.15–0.20)	0.95 (0.90–0.95)
T/S ratio	0.94 (0.76–1.12)	0.50 (0.48–0.55)	1.50 (1.44–1.54)
cT/S ratio	0.89 (0.59–1.27)	0.33 (0.32–0.37)	2.40 (2.05–2.63)

## Data Availability

Dataset available upon reasonable request from the authors.

## Supplementary Materials

The following supporting information can be downloaded at: https://www.mdpi.com/article/10.3390/ani15101495/s1, Table S1. Pre-treatment signalment, serum thyroid and creatinine concentrations, thyroid scintigraphic findings, and radioiodine dose in 234 ^131^I-treated cats, divided into three thyroid outcome groups.

## Abbreviations

The following abbreviations are used in this manuscript:

^131^I	Radioactive iodine
T4	Thyroxine
TSH	Thyroid-stimulating hormone
TcTU	Percentage thyroid uptake of 99mTcO_4_^−^
T/S	Thyroid-to-salivary ratio
cT/S	Corrected thyroid-to-salivary ratio
^99m^TcO_4_^−^	^99m^Tc-pertechnetate
ROI	Region of interest
IQR	Interquartile range
MBq	Megabecquerel
mCi	Millicurie
SHIM-RAD	Severe hyperthyroidism, Huge thyroid nodules, Intrathoracic tumor location, Multifocal distribution of radionuclide uptake, and Refractory to treatment with to Antithyroid Drugs
