# Supplementary material for: Thyroid Scintigraphy Findings in 234 Hyperthyroid Cats Before and After Radioiodine Treatment
Source: Animals (Basel). 2025 May 21;15(10):1495. doi: 10.3390/ani15101495 (PMC12108284; doi:10.3390/ani15101495)
Supplement: Supplementary file 1 [file animals-15-01495-s001.zip › Table S1.pdf]

Table S1: Pretreatment signalment, serum thyroid and creatinine concentrations, thyroid scintigraphic findings, and radioiodine dose in 234 ^{131}I-treated cats, divided into in 3 thyroid outcome groups

Variable	All cats (234)	Euthyroid (165)	Overt Hypothyroid (15)	Subclinical Hypothyroid (54)	*P* value
Age (years)	12 (11-14)	12 (11-14)	14 (12-16)	12 (10-14)	.099
Breed (mixed: purebreed ratio)	213:21 (10.1)	151:14 (10.7)	14:1 (14.0)	48:6 (8.0)	.848
Sex(female:male ratio)	131:103 (1.27)	78:87 (0.90)[ab]	14:1 (14.0)[a]	39:15 (2.6)[b]	<.0001
Serum creatinine (mg/dL)	1.0 (0.8-1.3)	1.0 (0.8-1.2)	1.2 (0.8-1.8)	1.0 (0.8-1.3)	.281
Serum T4 (µg/dL)	9.2 (6.6-12.4)	9.2 (6.6-12.7)	8.3 (5.3-12)	9.5 (6-12.4)	.620
Serum TSH (ng/mL)	0.02 (0.02-0.02)	0.02 (0.02-0.02)	0.02 (0.02-0.04)	0.02 (0.02-0.02)	.082
Detectable serum TSH concentration	8 (3.4%)	4 (2.4%)	2 (13.3%)	2 (3.7%)	.093
Bilateral:unilateral nodule	135:99 (1.36)	91:74 (1.23)	9:6 (1.5)	35:19 (1.84)	.447
Asymmetric:Symmetric bilateral disease	108:27 (4.0)	74:17 (4.35)	6:3 (2.0)	28:7 (4.0)	.487
Thyroid:salivary (T/S) ratio	5.3 (3.3-9.0)	5.5 (3.3-9.1)	4.5 (2.9-6.3)	5.3 (3.6-8.9)	.575
Percent thyroidal uptake of ^{99m}Tc-pertechnetate (TcTU)	4.3 (2.6-10.5)	4.0 (2.2-8.7)	3.6 (2.3-15.1)	6.9 (3.4-11.7)	.201
Dose radioiodine (mCi)	2.0 (1.8-2.5)	2.0 (1.8-2.5)	2.0 (1.8-3.4)	2.0 (1.85-2.5)	.735

Continuous data (age, serum concentrations of T4, TSH, and creatinine, T/S ratio, TcTU, and radioiodine dose) are expressed as median (25th-75th percentile) and analyzed with the Kruskal-Wallis test, followed by Dunn multiple comparisons test. Qualitative data are expressed as ratio (breed, sex) or number (%) of cats (detectable TSH concentration, bilateral: unilateral nodule) and analyzed with the Fisher's exact test, followed by the Holm-Bonferroni correction procedure for within group comparison. Values with the same superscript letters are significantly different to one another. Reference intervals: T4 = 1.0-3.8 µg/d; TSH = <0.03-0.3 ng/mL; T/S ratio = 0.5-1.65; TcTU = 0.2-0.95% (see Table 1).
